# Futures of gastric cancer: The fundamental level of prevention in health promotion using casual layered analysis

**DOI:** 10.1016/j.heliyon.2024.e40437

**Published:** 2024-11-15

**Authors:** Hassan Reyhani Khouzani, Mohammad Reza Maleki, Ali Zackery, Ehsan Mazloumi, Mohsen Jalilzadeh, Mandana Sahebzadeh

**Affiliations:** a Department of Health Services Management, School of Health Management and Information Sciences, Iran University of Medical Sciences, Tehran, Iran; b Department of Industrial Engineering and Futures Studies, Faculty of Engineering, University of Isfahan, Isfahan, Iran; c Health Management and Economics Research Center, Isfahan University of Medical Sciences, Isfahan, Iran

**Keywords:** Gastric cancer, Futures studies, Causal layered analysis, Deconstruction

## Abstract

Gastric cancer is the leading cause of cancer mortality among men and the second leading cause among women in Iran. Given the high incidence and mortality rates of this disease in the country, a deeper investigation into its effective causes is essential. One effective approach to uncovering the unknowns related to gastric cancer is the application of critical-deconstructive future-thinking tools, particularly Causal Layer Analysis (CLA). This qualitative study involved a review of theoretical foundations and meta-documents, along with interviews with a group of experts. By employing triangulation, the findings from the literature review were integrated with thematic analysis of the interviews through the CLA framework. At the litany layer, gastric cancer is identified as the second most prevalent and deadly cancer in Iran. The systemic layer explores the "social, technological, economic, environmental, and political" origins of gastric cancer, highlighting factors such as globalization, governance weaknesses, and cultural shifts. The third layer challenges the conceptualization of cancer as a metaphysical sin, advocating for a new narrative centered on Fundamental Prevention. Effective cancer control should focus on preventing the emergence and institutionalization of factors at the worldview and metaphorical levels that contribute to economic, political, social, and cultural attitudes, ultimately manifesting in high-risk behaviors and pathogenic processes. Designing complex social nudges is crucial for establishing this new narrative based on fundamental prevention. This study effectively combines a systematic literature review and semi-structured interviews to investigate the factors influencing gastric cancer in Iran through Causal Layer Analysis (CLA) and proposes a new layer to the conventional three-pronged prevention model called "fundamental level prevention." The findings suggest that interventions targeting worldviews and cultural beliefs can promote behavior change and enhance health outcomes, particularly among marginalized groups. Furthermore, prioritizing fundamental changes at these layers can lead to positive reductions in disease incidence. This research holds significant implications for international audiences, highlighting the complexities of gastric cancer and the necessity for cultural interventions and global collaboration to address this pressing health issue.

## 1 Introduction

Cancer is a large group of diseases in which the main common denominator is the irregular and unrestrained growth of cells which can lead to the disruption of the body's natural balance and serious and deadly disorders [1,2]. Today, more than 200 types of cancer have been identified and its prevalence is increasing; about 20 million people worldwide are currently battling the disease (excluding 17 million skin melanoma cancers). At present, cancer is the second leading cause of death in developed countries and the third leading cause of death in developing countries [3,4].

Gastric cancer is one of the most common and deadly cancers, especially among the elderly. Gastric cancer with an annual incidence of about 1,033,701 cases is about 5.7 % of the incidence and with 782,685 deaths is one of the first deadly cancers. According to the GLOBACAN2018 report, gastric cancer is in second place with an annual incidence of 11.644 cases, about 10.6 %; additionally, gastric cancer is the fourth most common cancer and the second leading cause of death in the world [5].

Gastric cancer is a leading cause of death from gastrointestinal diseases in Iran [6], driven by factors such as dietary habits, genetic predispositions, and environmental influences [4,5,7,8]. Key contributors include high salt intake, processed food consumption, and *Helicobacter pylori* infections. Cultural and socioeconomic factors often lead to late diagnoses, as many patients seek help only in advanced stages, exacerbated by a lack of screening programs and public awareness.

To effectively address this issue, it is crucial to identify the underlying causes of gastric cancer. This study employs "Causal Layered Analysis" (CLA), a holistic method that explores various levels of contributing factors [[9], [10], [11]]. Unlike previous research that focused on visible causes, this approach aims for a deeper understanding of the drivers behind gastric cancer, highlighting the need for targeted interventions and comprehensive research to combat this significant health challenge in Iran.

## 2 Literature review

The ever-increasing importance of cancer as a leading cause of death has resulted in cancer being investigated by various disciplines including but not limited to psychological, sociological, economic, anthropological, and interdisciplinary perspectives. In what follows, we will briefly look at the contribution of some of these works [12,13].

Dhavan Shah studied the environmental and genetic risk factors for gastric cancer and reported that "smoking, diet, and environmental exposure" were factors in the systemic layer as causes of cancer [14]. Another study by Yang Ging Lin et al., in 2018 looked at factors such as "age, sex, smoking, low socioeconomic status, salt intake, and smoked food, low consumption of fruits and vegetables, obesity, physical activity, as well as regular screening by the health care system for high-risk groups, eradication of *Helicobacter pylori*," and their contribution to gastronomic cancer [15]. From a CLA perspective, this study investigates some systemic factors but does not go deeper. Similarly, Catlan et al. showed that socio-economic factors and the incidence of stomach cancer have a significant correlation; the direct relationship between the unemployment rate, household size, and the inverse relationship between literacy rate, urbanization ratio, and household cost to cancer incidence was reported in this study [16]. Findings from Nolan et al. in a paper entitled "Gastric Cancer in Alaska Native and American Indian People Living in Alaska, 1990–2017 ″ based on the determining role of lifestyle factors at risk of gastric cancer confirms the importance of socio-economic factors as well [17]. As for screening, a 2016 study—a psychological one— by Lang et al. found no link between fate and fear of cancer and screening. They report that “there were no significant associations between fatalism and cancer fear with screening” [18].

The research background about stomach cancer contains points that show the relationship of this disease with economic and social parameters and lifestyle. The direct relationship between the incidence of this disease and the unemployment rate, the size of the household, and the reciprocal relationship between the literacy rate, the ratio of urbanization, and household expenses are evident in the study of Arjalo et al. (Heidari N,2015). Additionally, in the study of Delfan Biranv and Rostami, the environmental, social, and economic conditions of the residential area are also mentioned as one of the factors that may contribute to stomach cancer [3,19]. The results of Elizabeth Ward's study indicate that the death rate of cancer in deprived provinces and regions is 31 % higher than in prosperous regions [20]. Analogously, male gender, environmental factors, smoking, increasing age, diet, and lifestyle have been listed as risk factors for stomach cancer [21]. The results of this study were confirmed in another study conducted by Adam Barsouk; in this research, socio-economic and lifestyle factors such as smoking, alcohol, chemical exposure, diet, obesity, gender, and race are found to be effective in the proliferation of stomach cancer [5].

CLA has also been used by some researchers to identify the causes of some diseases to provide a fresh deeper perspective on the hidden dimensions of health-related issues [9,22]. In a participatory study using civic epistemologies and CLA theoretical apparatus, Kewulay Kamara tried to elicit the stories of Ebola in 2064 from the audience [22]. At a deeper and broader level, Rhyll Vallis and Sohail Inayatullah investigated the governing policy metaphors of tuberculosis and obesity and conclude that these metaphors dictate a certain manner of problem conceptualization and formulation of solutions [22]. Another striking study of this ilk is a study conducted by Robinson et al., in which the authors use CLA to systematically review the literature on occupational therapy services for people with chronic pain to deconstruct conventional approaches and provide a fresh perspective [23]. Despite these works, most studies conducted about the causes of cancer investigate the litany and systemic causes of cancer. In this paper, we intend to provide a deeper perspective of the origins of cancer by using CLA.

## 3 Materials and methods

This paper uses CLA to systematize and deconstruct how gastronomic cancer is conceptualized and treated to help policy formulations concerning this type of cancer in particular and cancer in general.

Inclusion and exclusion criteria for meta-documents included a review of articles and high-level documents related to political and health systems that specifically focused on aspects of cancer prevention and treatment. These documents had a significant impact on health policies and cancer control programs in the country. Additionally, to select the interviewees, individuals with scientific or professional experience in the field of cancer were chosen, ensuring that they had at least a few years of relevant experience. This choice was made to ensure the depth and validity of the opinions collected.

**Fig. 1:**
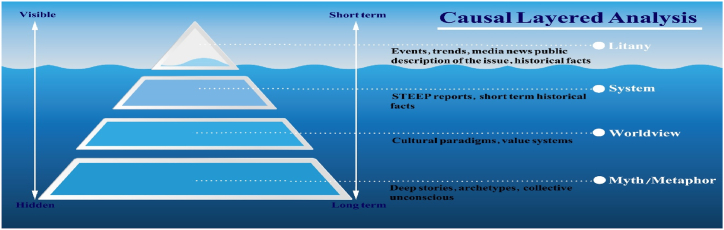
CLA layers different layers of causal layered analysis.

Our analysis reveals that critical post-structural approaches can provide a fresh perspective concerning how cancer should be tackled not as merely a health anomaly but as a multidimensional phenomenon. CLA examines the causes of the subject at four levels as shown in Fig. 1 [24].

The opinion of 20 experts was obtained, which includes people who have scientific knowledge or professional experience or both in the field of cancer. The characteristics of the experts are shown in Table 2.

**Table 1:** Findings resulting from the literature review and interviews.

Layer examined	Findings resulted from a systematic literature review of the pertinent literature and thematic analysis of Upstream Documents	Findings from Interviews
**Litany**	Cancer tsunami; Gastric cancer is the deadliest cancer in Iran; The highest incidence of cancer in Iran with an annual incidence of 10,500 new cases; The most common cancer in Iranian men; One of the 5 most common cancers in Iran(1–3, 5)	
**System**	**Social** •The literacy level of individuals (2–4) •Family consolidation status •Smoking (4, 6) •Alcohol consumption •Addiction (1–6) •The trend of an aging population (1–6) •Type of nutrition(1–6) •Lifestyle (1–6) •Physical activity status and sedentary lifestyle (1–6,21) •Lifestyle development derived from the Western culture (1–6, 21–23) •Eating habits (1–6,21-36) •Food processing and storage style (1–6, 21–36)	**Social**: •Population increase (P_1,_ P_4_) •Change in family patterns (P1-P7) •Type and style of construction of the city (residential, commercial complexes, etc.) (P_2_) •Mediatization of lifestyles and the role of celebrities/influencers/social networks (pP3-P8) •The psychological impact of the mismatch between the facilities and expectations of society (P_1-_P_3_) •Declining mobility of social status about employment and education (P_9-_P_11_) •Decreasing employment relationships (job satisfaction and security); the phenomenon of the working poor (P_8-_P_10_) •Mechanization of life (P_19-_P_21_) •The growing trend of the speed of life (P_3,_ P_19_) •Increased social stressors and social tensions (P_5,_ P_13)_ •Increased risk-taking: the growth of high-risk behavior in Iran (P_9,_ P_25,_ P_26_) •Inadequacy of training about risk reduction and stress coping mechanisms (P_1,_ P11) •Lack of appropriate alternatives to reduce risk-taking or risky behaviors (P19, P24) •Decreasing social vitality (P13,-P15) •The rising trend of migration to metropolitan areas i.e., urbanization (P7-P11) •Population aging (P2-P15) •Disintegration of families (P14-P19) •Changing the attitude and culture of society concerning the social status (P9, P13, P18) •Consumerism and conspicuous consumption (P7, P18, P23) •Fluid Gender roles (norms of men and women) (P19) •The growing trend of changing the values and norms of nutrition style towards easy and fast nutrition and mixing (Eating several types of food and drinks in one meal) (P21-P23) •The rise of individualism (P26)
**System**	**Technology** •Industrialization of societies (42, 43)	**Technology** •The growing trend of industrialization (P8, P15, P19) •The increasing mechanization of life (P16, P19) •The entry of new pollutants into the environment (P6, P11, P26)
**System**	**Economic** •Childhood poverty (36, 38) •Family income levels (34, 35) •Employment status of the breadwinners of family (33–35) •The economics of drug and tobacco (30, 33–35)	**Economic** •Globalization (P3, P8, P9) •Export of single products (P8, P9) •Heavy dependence on oil exports (P11, P18) •The growing trend of poverty (P5, P13, P24) •Inflation and super-inflation (P13, P18, P25) •Decrease in the value of Rials against other currencies (decrease in purchasing power) (P15-P19) •Increasing trend of economic and social class divisions (P1-P3, P19) •Nutrition welfare and related issues such as obesity and metabolic syndrome (P18-P23) •Dependence on some strategic and non-strategic products (P6-P10) •Import of transgenic products (P1-P3, P17) •Rising unemployment (P16) •An extremely high turnover rate of tobacco and narcotics (P18, P21)
**System**	**Environmental** •Environmental pollution (33) •Pollution of water resources (33) •Soil pollution (28, 33) •Air pollution(28, 33) •Excessive use of chemical fertilizers and pesticides (28, 29) •Use of preservatives (26, 28, 29) •Pollutants from low-quality fuel consumption or incomplete combustion in vehicles (28, 29, 33) •	**Environmental** •Lack of a specific trustee in water, air, and food management (pollution is the silent killer) (P3, P7, P11) •Increase in environmental pollution (P8, P15, P19) •Environmental pollution in poor areas (P5, P13, P126) •Unusual use of chemical fertilizers and pesticides (P6, P16) •Increase in consumption of permitted and unauthorized preservatives and additives (P2, P18, P21) •Mass production policies (P4, P10, P17) •Inadequate supervisory mechanisms of the health of food production processes and distribution chains (P9, P16, P26)
**System**	**Political** •Lack of upstream health legislation, especially for cancer (1–6, 18–21) •Decreased priority of environmental protection during the implementation of macro-industrial projects despite the emphasis on laws (18–21)	**Political** •Non-governmental nature of governance (existence of guiding dominates political currents and informal networks in the country) (P5, P9) •The rule of the principle of "order in disorder" in the country or the absence of serious rules; Island policy-making in the country (P5, P18) •Limited use of the country's existing legal leverages; legal vacuum related to cancer issues (P9, P15) •Free supply of tobacco products (lack of restrictive laws) (P3, P19, P25) •Lack of health-oriented policy in the country (P7, P16) •The Discourse of the Islamic Revolution (P13, P18-P21) •International relations(P8, P11) •Foreign sanctions (P19-P24) •Internal political relations (P1, P9, P23) •Poor implementation of existing laws in the field of soil, water, food, and air security to provide adequate and healthy food (P8-P14) •Insufficient attention to the development and implementation of prevention and screening programs for gastric cancer (P6-P9)
**System**	**Health drivers** •The second priority of health and prevention at the micro and macro level is in the eyes of the country's policymakers and stakeholders (33).	**Health drivers** •Priority of treatment over health (P3-P11) •Insufficient budget of the Ministry of Health (P8-P12) •Incidence of emerging and re-emerging diseases (P17-P19) •Increasing legal and illegal health expenses in the household dimension (out-of-pocket payments, etc.) (P1, P8-P10) •Insufficient attention to aging health policies (P17, P21-P24) •Lack of comprehensive health policy (P7, P19, P23) •Achieved and expected successes in cancer treatment (P6-P9) •Inadequate protein food substitutes (P14, P17, P123) •Occupational Carcinogenic Risks (P14, P17, P26)
**Worldview and discourse**	•Fatalism discourse (17) •Religious beliefs about salt consumption (17) •Clash of modern medicine and traditional medicine (17)	•Lack of responsibility concerning personal health (P8, P25) •Conceptualization of cancer as a meta-physical punishment caused by sins (P3,P17, P24) •Justice-oriented ideology vs. free market ideology in health policy (P18, P22, P25) •Superstitious traditional medicine discourse advertised by hardliners (P14, P19) •Mac-Donaldism (P11, P18)
**Metaphor and myth**	•Cancer is always a death sentence(17) •Cancer is a "monster" that lurks within the body, growing and spreading until it takes over(18) •Institutionalizing proverbs in people's minds like "There is no mortal food"	•The conceptualization of cancer as a battle or war to be won(P1, P8, P16, P21) •God is bigger than my cancer(P6, P13, P19) •Cancer is God's plan(P8-P11) •Cancer is an "enemy" or "invader" that must be fought and defeated(P4, P13, P124) •“I am a super-man”— Cancer will not visit my door (P6, P15, P23, P26).

**Table 2:** The characteristics of the experts.

Number	University	Field of study
1	Professor of Isfahan University	PhD in Future Studies
2	Professor of Shahid Beheshti University of Medical Sciences	PhD in Future Studies
1	Professor of Isfahan University of Medical Sciences	PhD in Health Economics
2	Professor of Isfahan University of Medical Sciences	PhD in Social Health and Welfare
1	Professor of Isfahan University of Medical Sciences	PhD in Health Policy
1	Professor of Isfahan University of Medical Sciences	PhD in Health Information Technology
1	Professor of Isfahan University of Medical Sciences	PhD in Health Management
1	Professor of Isfahan University of Medical Sciences	PhD in Elderly Health
1	Professor of Isfahan University of Medical Sciences	PhD in Nutrition
2	Professor of Isfahan University of Medical Sciences	PhD in Environmental Health
1	Professor of Isfahan University of Medical Sciences	PhD in Health Education
2	Professor of Isfahan University of Medical Sciences	PhD in Health Economics
2	Professor of Isfahan University of Medical Sciences	PhD in Persian Literature
2	Activities in the private and public sector	physician

The reviewed documents include a superficial review of articles and high-level documents related to the political and health systems (as explained in the text). High-level documents that were not available have been removed.

Using the guide and semi-structured questionnaire, interviews were conducted.The interviews lasted between 30 and 45 min, and most of them took place in the individuals' offices or workplaces. Coordination initially occurred over the phone, after which the questionnaire was sent to the individuals. In the next stage, the interviewers conducted face-to-face interviews with the participants, and based on the participants' answers, more depth was added to the interviews.

Specialists were individuals with a scientific or professional background who oversee the research subject in systemic, worldview, and metaphorical dimensions.

The layer of objective or litany issues observes the most objective level of the future, and its understanding does not require special analytical skills. In this layer, the occurrence of the future is accepted by everyone according to the quantity of data and information that is published in the public media.

The social cause layer looks at economic, cultural, political, and historical factors. Litany data and information are described and questioned at the second level.

At the third level, deeper hypotheses, reasoning, worldviews, and ideologies are identified. This layer oversees the understanding of the structure, worldview, and discourse that not only supports future epistemic claims but also legitimizes these claims and forms the causes of the objective problem on the first layer.

The fourth layer expresses the emotional dimensions of the unconscious and seeks to understand the signs and metaphors that, subconsciously, form the dominant worldview and discourse that give rise to social causes and objective problems [9,25,26].

**Fig. 2:**
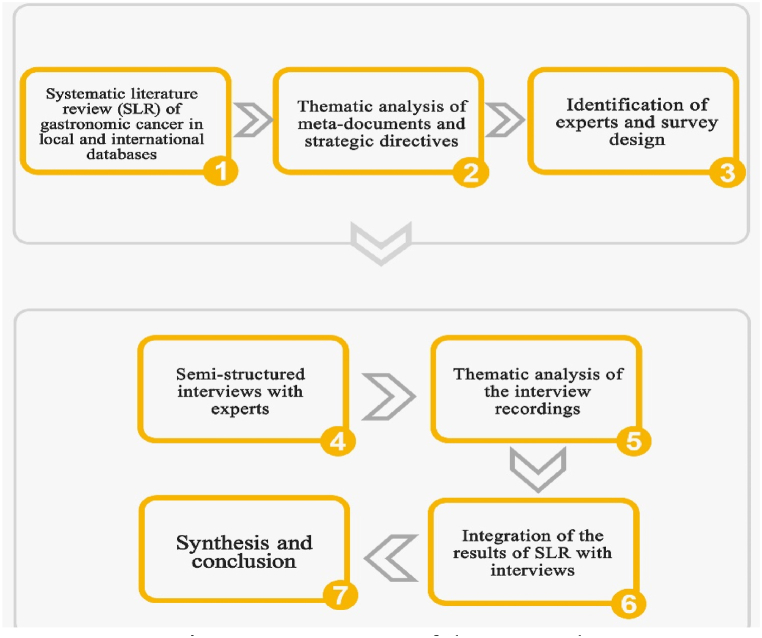
Process steps of the research.

As Fig. 2 illustrates we conducted the research through method triangulation to create a holistic picture of the phenomenon under study. In the first step, to identify the causes of gastric cancer in different layers of CLA, library sources and meta-documents were examined. Library resources included a review of articles, research, and texts related to gastric cancer in databases inside and outside Iran, including Magiran, Normex, google scholar, PubMed, ScienceDirect, and Web of Science. For this purpose, various keywords were used, including cancer, gastric cancer, stomach cancer, gastric neoplasm, stomach neoplasm, colorectal cancer, risk factors for gastric cancer, and socioeconomic factors of gastric cancer for internal and external sources.

In the second step, the review of the latest documents related to the prevention and treatment of cancer in general and gastric cancer in particular in the political, legislation, health, and other related systems of Iran was performed.

The meta-documents used had two characteristics of permanence and impact on the long-term horizon of the country, which included: “constitution of the country, five-year development plans, announced policies of the Supreme Leader, comprehensive scientific plans, comprehensive health plan, comprehensive cancer control programs, and health system transformation plans ”. The output of this step was used to provide a framework to compile the interview guide to be used in the following steps as well as the initial completion of different layers of CLA.

In the third step, a questionnaire was designed to be used in semi-structured interviews. At the global level, the SLR we conducted gave us a broad picture of the antecedents of gastronomic cancer but we decided to incorporate semi-structured interviews into the research process to create a more accurate picture at the local level. To increase the validity of the questionnaire, experts' opinions were sought. Corrections were made according to experts’ recommendations to compile a complete and comprehensive questionnaire based on CLA layers. This was followed by the expert identification phase. As suggested by Andersen et al. [27]. these stakeholders were included to widen the peripheral vision of the research team and increase the depth of antecedents of gastric cancer in Iran based on CLA theoretical lens. In this regard, experts are people who have academic education or executive experience in their field or both. The semi-structured interview guide was prepared based on the results of the first step of the study and was reviewed and approved by the research team after conducting several preliminary interviews. The interview guide was sent to the experts with prior coordination, and after reading the interview guide, an interview was held at the appointed time to obtain the comments of the person in the interview. In all steps of the study (from data collection to analysis and reporting of findings), items such as informed consent, anonymity, confidentiality of information, and the right to leave the study at any time and record interviews were considered. After that, all recorded interviews were implemented and the information was carefully studied for further analysis. The accuracy and robustness of the collected information were assessed using the four Guba and Lincoln criteria including data acceptance, reliability, validation, and transmission capabilities [28].

**Fig. 3:**
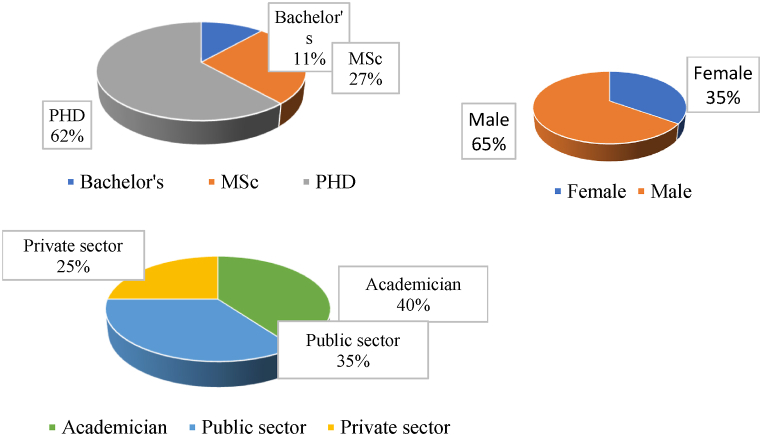
Demographic features of experts.

The average duration of each interview was half an hour. Fig. 3 shows the demographic features of the 26 experts who were interviewed. Experts were chosen from the pool of researchers we had created while doing the initial environmental scanning, health system managers, and independent researchers through snowballing and co-nomination [27].

The group of experts in this research had one of the following two characteristics: academic education and executive experience related to the fields of interest in the research.

For the final analysis, the findings from the interviews were organized according to the subcategories of the Causal Layer Analysis (CLA) layers. The collected opinions, which often included epidemiological data along with incidence and prevalence indicators, were categorized under the litany layer. In the systemic layer, we compiled various subcategories based on the STEEP analysis, which encompasses "social, technological, economic, environmental, and political" domains. Additionally, health-related factors were considered separately, leading to the classification being defined as STEEPH.

In the worldview and metaphor layers, we followed a similar approach, gathering the categories and subcategories relevant to each layer.

To ensure the validity and accuracy of the research, we examined the credibility, dependability, confirmability, and transferability of the data. This process involved collecting authentic data and increasing the number of interviews to enhance accuracy. We also presented the findings to the participants for validation and to evaluate their experiences.

To ensure transferability, we employed the maximum diversity sampling method. For internal validity, the interviewer refrained from imposing their opinions on the participants, fostering an informal relationship to create a comfortable environment that encouraged open expression of opinions. The study utilized member checking and repeated questioning to identify any contradictions in participants' responses.

To ensure dependability and confirmability, a member of the research team independently analyzed a subset of the interviews, resolving any coding disagreements with input from a third party. Finally, we recorded, transcribed, and thematically analyzed the interview recordings. We then integrated the results of this thematic analysis with findings from previous stages using the different layers of CLA.

## 4 Results

The findings of this study are presented in two parts according to the steps of its implementation. The findings from the thematic analysis of the upstream documents, the literature review, and the interviews were combined based on layers of the CLA. The thematic analysis of the semi-structured interview is presented separately to provide us with an opportunity to compare the results of these two procedures. Table 1 shows the findings of this study based on CLA Layers.

## 5 Discussion

In the literature reviews, the findings of this study are consistent with the results of other studies in the system layer. In some studies, "diet, lifestyle, demographic characteristics such as age, gender, and occupational exposure" have been emphasized [29]. Some other studies have found the role of environmental factors such as "tobacco consumption, diet, and environmental exposure" to be effective in the occurrence of cancer [30]. Likewise, factors such as age, gender, smoking, low socio-economic status, salt and smoked food consumption, low consumption of fruits and vegetables, obesity, physical activity, and regular screenings by the health care system for high-risk groups Eradication of *Helicobacter pylori*" are mentioned [31].Also, in this regard, the direct relationship between the unemployment rate, the size of the household and the reverse relationship between the literacy rate, the ratio of urbanization and the household expenditure to the incidence rate of cancer was evident in other studies. Above all else, the determining role of lifestyle factors in the occurrence of this cancer has been considered [32]. which was one of the recurring themes in the interviews (See Table 1).

Nonetheless, the findings of this study in the layers of worldview, and metaphor are original, innovative, and striking in comparison to other studies. Numerous economic problems, huge class distinctions stress and tensions, the silent killer of pollution, health system policies neglecting the prevention and promotion of public health and paying more attention to treatment, wrong customs and traditions, and misconceptions are all factors that can affect the future of gastric cancer in Iran.

This research attempted at collecting some hidden dimensions of gastronomic cancerin Iran. Of course, the findings are based on the historical, cultural, and civilizational patterns of Iranian society, and can be different or similar in other societies based on its historical, cultural, and ideological patterns. The first issue that can be mentioned is that the causes of gastric cancer in the long-term horizon of Iranian society are many, complex, and intertwined. Another point is that according to the factors mentioned before in different layers, especially the systemic layer, any comprehensive planning in the category of cancer prevention, especially gastric cancer, should be based on special attention to metaphorical layers, worldview and drivers in the systemic layer, especially in economic, social and cultural policy-making; in other words, among these layers can be found factors that not only do not act as an obstacle to the implementation of programs and policies of the health system but can also be an opportunity for good factors to better implement these policies. Another important issue that showed its importance in this study is the issues related to prevention levels. So far, levels of prevention have been defined in four levels including primordial, first, second, and third levels, the deepest of which is related to the primordial level of prevention (see Fig. 3); primordial prevention includes activities and actions that minimize health risks and thus prevent the emergence and establishment of processes and factors (environmental, economic, social, behavioral, and cultural) that have been shown to increase the risk of disease. The purpose of primordial prevention is to prevent the emergence and establishment of those social, economic, and cultural patterns of life, whose role in increasing the risk of various diseases is known [33,34].

As defined, the primordial level prevention focuses at best on environmental, economic, social, behavioral, and cultural factors; In fact, it ultimately covers the causes up to the systemic level, while it lacks any focus on the levels of worldview and metaphor. Here, due to the influence of factors related to the two layers of worldview and metaphor on the upper layers, a new level of prevention is proposed by this study called fundamental prevention. This level prevents the emergence and institutionalization of factors that at the worldview and metaphorical levels of societies cause economic, political, social, and cultural attitudes that occur in behaviors, patterns, and high-risk pathogenic processes. If one considers the proliferation of cancer as the leading cause of death in Iran as a developing society, the way its conceptualized as a meta-physical sin or the way a divine narrative hovers around it, has a huge impact on the current status of gastronomic cancer in Iran and how it should be tackled in future. The fundamental prevention, therefore, calls for crafting a new narrative for the way cancer is conceptualized. It calls for a secular and socio-economic image of cancer which is caused by our lifestyles, eating habits, and genetic predispositions. More importantly, cancer can also be cured if it is diagnosed on time and the necessary steps are taken promptly. Fundamental prevention also calls for health to be conceptualized as an “absolute right” and not a “luxurious commodity” which in turn necessitates measures taken to create socially impactful systems and subsystems. In this new narrative, instead of screening programs, availability of cancer therapy, and/or cancer insurance coverage, prevention-based human-oriented macro settings are strategically sought to change “what we eat”, “what we inhale”, “how we commute”, “how much stress we endure and for how long”, and “how valuable our health is considered”.

Fundamental prevention refers to strategies and interventions aimed at addressing the root causes of a particular health issue or condition, to prevent it from occurring in the first place. It involves targeting the underlying factors that contribute to the development of a disease or condition, rather than simply treating the symptoms or consequences. Overall, fundamental prevention, which involves addressing the underlying factors that contribute to health outcomes, is an important strategy for promoting health and preventing disease. By addressing the social determinants of health, policymakers and public health practitioners can help to create environments that support healthy behaviors and improve health outcomes in the long term. Fundamental prevention may involve a range of strategies, including.

1. Health promotion: This includes education and awareness campaigns aimed at promoting healthy behaviors and lifestyles, such as regular exercise, healthy eating, and avoiding tobacco and alcohol.
2. Environmental interventions: This involves modifying the physical and social environment to promote healthy behaviors and reduce exposure to harmful factors, such as improving access to healthy food options and safe recreational spaces.
3. Policy and regulatory interventions: This includes policies and regulations aimed at reducing exposure to risk factors and promoting healthy behaviors, such as taxes on tobacco and alcohol, regulations on food marketing to children, and workplace policies that promote physical activity.
4. Socioeconomic interventions: This involves addressing the social determinants of health, such as poverty and inequality, which can contribute to the development of health issues. This may include interventions aimed at improving access to education, employment, and affordable housing.
5. Worldview intervention
Worldview intervention refers to a type of intervention aimed at addressing the underlying beliefs, values, and attitudes that shape an individual's worldview. This type of intervention recognizes that people's worldviews can have a significant impact on their behaviors and choices, particularly in the context of health and well-being.
6. Metaphor and myth intervention

Metaphor and myth interventions refer to a type of intervention that uses storytelling, metaphor, and myth to promote behavior change and address underlying beliefs and attitudes. This type of intervention recognizes that stories and myths can have a powerful impact on an individual's worldview and can shape their beliefs and behaviors.

Overall, metaphor and myth interventions recognize that behavior change requires more than just providing information or incentives. Rather, it requires engaging individuals on an emotional and symbolic level and using storytelling and myth to promote positive attitudes and beliefs that support behavior change.

**Fig. 4:**
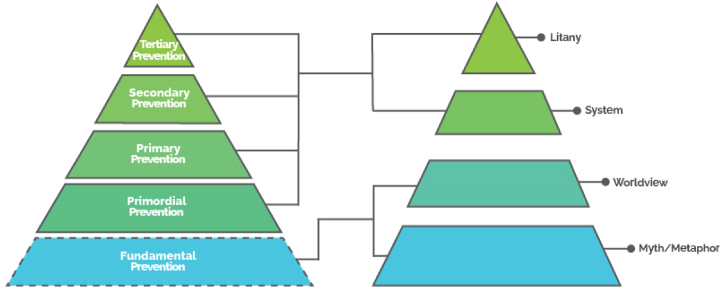
The conceptualization of fundamental-level prevention.

This level of prevention deals with issues related to prevention at a deeper and more comprehensive level and explores the causes of diseases at the lowest, deepest, and most comprehensive levels; this level of prevention invites human society to reconsider its self-made values, superstitions, and ineffective metaphors (Fig. 4). This level of prevention requires modernity along with a positive view of valuable traditions and avoidance of their troublesome extras.

Integrating insights from various studies can significantly enhance our understanding of the complex factors influencing gastric cancer in Iran. For instance, one study emphasizes the importance of addressing the multifaceted nature of health issues through a layered approach, which can unveil the underlying beliefs and values that shape societal responses to cancer [35]. This layered analysis allows researchers to identify not only the symptoms of the issue but also the deeper cultural narratives and social norms that influence health behaviors and perceptions [35].Similarly, another study illustrates how cultural and economic dimensions can deeply affect public health outcomes, highlighting the need for tailored interventions that resonate with local contexts [36]. These interventions must consider the unique socio-economic landscape of Iran, including factors such as access to healthcare, educational disparities, and cultural attitudes toward illness and treatment. By acknowledging these dimensions, health initiatives can be more effectively designed to engage the community and promote healthier behaviors [36].Furthermore, several studies showcase the effectiveness of examining systemic and metaphorical layers in designing interventions that are not only practical but also culturally sensitive [37]. This approach ensures that health programs are developed in collaboration with local communities, thus fostering trust and cooperation. By integrating local knowledge and practices, these interventions can achieve greater acceptance and success.Collectively, these studies underscore the necessity of employing a comprehensive framework that considers both the visible and hidden factors contributing to health challenges. This holistic perspective is crucial for understanding the complexities surrounding gastric cancer in Iran and ultimately guiding more effective prevention strategies. By addressing the root causes of health issues and engaging with the community's cultural context, public health initiatives can lead to sustainable improvements in health outcomes and quality of life for individuals affected by gastric cancer.

## 6 Conclusion

This study combined a systematic literature review with semi-structured interviews to create a comprehensive understanding of the drivers of gastric cancer in Iran through Causal Layer Analysis (CLA). We introduced an additional layer to the traditional three-pronged prevention model, termed "fundamental level prevention." This new level encompasses health promotion, environmental and socio-economic interventions, as well as worldview and myth-based prevention strategies.

Our findings suggest that interventions addressing worldviews, which consider cultural beliefs and values, can significantly promote behavior change and enhance health outcomes, especially among marginalized or underserved populations. Moreover, integrating metaphor and myth interventions with educational and awareness campaigns can further drive effective behavior change.

Fundamental prevention is crucial for health promotion and disease prevention, as it tackles the underlying factors contributing to health issues. Therefore, efforts to combat gastric cancer and similar diseases should prioritize worldview and metaphorical factors. By modifying risk factors at these levels, we can instigate fundamental changes in the occurrence and emergence of such diseases. Positive changes in these foundational layers can lead to beneficial ripple effects throughout other layers and address the root causes of the disease.

The implications of this manuscript are significant for international readers, particularly regarding the multifaceted influences on gastric cancer. By utilizing CLA and the STEEPH framework, our study offers a holistic perspective that transcends basic epidemiological data.

1. Holistic Understanding: The integration of social, technological, economic, environmental, political, and health-related factors reveals the complexity of gastric cancer's impact. This comprehensive approach invites international readers to consider how these layers interact across different cultural and geographical contexts.
2. Cultural Sensitivity: Highlighting the worldview and metaphor layers emphasizes the role of cultural narratives in shaping health perceptions and behaviors. This insight is essential for public health practitioners and policymakers worldwide, reinforcing the necessity for culturally tailored interventions in cancer prevention and treatment.
3. Methodological Rigor: The study's rigorous methods, including maximum diversity sampling and member checking, provide a model for future research in other regions. International researchers can adapt these strategies to enhance the credibility of their studies.
4. Practical Applications: The findings can guide the development of health communication strategies that resonate with diverse populations. Understanding the underlying beliefs and societal attitudes toward gastric cancer enables stakeholders to create more effective public health campaigns.
5. Global Collaboration: This manuscript advocates for collaboration among international researchers and health professionals to share insights and strategies for addressing gastric cancer on a global scale. The findings call for a collective effort to tackle the disease through a multifaceted lens, considering local contexts while learning from global experiences.

In summary, this manuscript not only enriches the existing body of knowledge on gastric cancer but also serves as a valuable resource for international audiences aiming to understand and address the complexities associated with this disease.

## CRediT authorship contribution statement

**Hassan Reyhani Khouzani:** Writing – review & editing, Writing – original draft, Methodology, Investigation. **Mohammad Reza Maleki:** Writing – review & editing, Writing – original draft, Methodology, Investigation. **Ali Zackery:** Writing – review & editing, Writing – original draft, Methodology, Investigation. **Ehsan Mazloumi:** Writing – review & editing, Writing – original draft, Methodology, Investigation. **Mohsen Jalilzadeh:** Writing – review & editing, Writing – original draft, Methodology, Investigation. **Mandana Sahebzadeh:** Writing – review & editing, Writing – original draft, Supervision, Project administration, Methodology, Investigation.

## Data availability

Data will be made available on request.

## Declaration of Competing Interest

The authors declare that they have no known competing financial interests or personal relationships that could have appeared to influence the work reported in this paper.
